# Does the impact of bereavement vary between same and different gender partnerships? A representative national, cross-sectional study

**DOI:** 10.1017/S0033291722000496

**Published:** 2023-07

**Authors:** Liadh Timmins, Alexandra Pitman, Michael King, Wei Gao, Katherine Johnson, Peihan Yu, Debbie Braybrook, Anna Roach, Steve Marshall, Elizabeth Day, Ruth Rose, Paul Clift, Kathryn Almack, Deok Hee Yi, Katherine Bristowe, Richard Harding

**Affiliations:** 1 Florence Nightingale Faculty of Nursing, Midwifery and Palliative Care, Cicely Saunders Institute, King's College London, London, UK; 2 Mailman School of Public Health, Columbia University, New York, NY, USA; 3 Division of Psychiatry, University College London, London, UK; 4 Camden and Islington NHS Foundation Trust, London, UK; 5 Social and Global Studies Centre, Royal Melbourne Institute of Technology, Melbourne, Australia; 6 King's College Hospital, London, UK; 7 Patient & Public Involvement, London, UK; 8 Patient & Public Involvement, Peacehaven, UK; 9 School of Health and Social Work, University of Hertfordshire, Hertfordshire, UK; 10 Department of Palliative Care Policy & Rehabilitation, King's College London, Florence Nightingale Faculty of Nursing, Midwifery & Palliative Care, Cicely Saunders Institute, Bessemer Road, London, UK

**Keywords:** Bereavement, sexuality, distress, LGBT, outcomes

## Abstract

**Background:**

Data suggest poorer bereavement outcomes for lesbian, gay and bisexual people, but this has not been estimated in population-based research. This study compared bereavement outcomes for partners of same-gender and different-gender decedents.

**Methods:**

In this population-based, cross-sectional survey of people bereaved of a civil partner or spouse 6–10 months previously, we used adjusted logistic and linear regression to investigate outcomes of interest: (1) positive screen on Inventory of Complicated Grief (ICG), (2) positive screen on General Health Questionnaire (GHQ), (3) grief intensity (ICG) and (4) psychiatric symptoms (GHQ-12).

**Results:**

Among 233 same-gender partners and 329 of different-gender partners, 66.1% [95% confidence interval (CI) 60.0–72.2] and 59.2% [95% CI (53.9–64.6)] respectively screened positive for complicated grief on the ICG, whilst 76.0% [95% CI (70.5–81.5)] and 69.3% [95% CI (64.3–74.3)] respectively screened positive on the GHQ-12. Same-gender bereaved partners were not significantly more likely to screen positive for complicated grief than different-gender partners [adjusted odds ratio (aOR) 1.56, 95% CI (0.98–2.47)], *p* = 0.059, but same-gender bereaved partners were significantly more likely to screen for psychiatric caseness [aOR 1.67 (1.02, 2.71) *p* = 0.043]. We similarly found no significant association of partner gender with grief intensity [*B* = 1.86, 95% CI (−0.91to 4.63), *p* = 0.188], but significantly greater psychological distress for same-gender partners [*B* = 1.54, 95% CI (−0.69–2.40), *p* < 0.001].

**Conclusions:**

Same-gender bereaved partners report significantly more psychological distress. In view of their poorer sub-clinical mental health, clinical and bereavement services should refine screening processes to identify those at risk of poor mental health outcomes.

## Background

Compared to non-bereaved individuals, bereaved people have significantly worse health prior to, and in the year following, bereavement and are significantly less likely to be employed up to 2 years post bereavement (Stephen et al., 2015). Those who lose a partner are less likely to access healthcare (Prigerson et al., 2001) and have increased odds of worsened or new onset physical illness (Thompson, Breckenridge, Gallagher, & Peterson, 1984) and death (Elwert & Christakis, 2008). A review of health outcomes among bereaved people concluded that in addition to affective, cognitive behavioural, physiological-somatic and immunological reactions to bereavement there is consistent evidence for early mortality, rates of disability mortality and hospitalisation. (Stroebe, Schut, & Stroebe, 2007)

LGBT+ people have higher smoking rates and recreational drug use, which are attributed to the experience of discrimination (Jackson et al., 2020; Mercer et al., 2016; Shokoohi, Salway, Ahn, & Ross, 2020). The relationship between minority stress and biological outcomes including a higher incidence of serious physical illness has been empirically established (Flentje, Heck, Brennan, & Meyer, 2020). Evidence suggests higher rates of certain cancers, (Quinn et al., 2015) such as breast and oropharyngeal cancer among cisgender lesbian or bisexual women, (Meads & Moore, 2013; Saunders, Meads, Abel, & Lyratzopoulos, 2017) cervical cancer among cisgender bisexual women, (Robinson, Galloway, Bewley, & Meads, 2017) and anal and penile cancer among cisgender gay or bisexual men (Machalek et al., 2012; Saunders et al., 2017). Same-sex cohabiting couples have higher mortality rates from cardiovascular and respiratory disease (Meads, Martin, Grierson, & Varney, 2018). Partners in same-sex relationships, therefore, have a greater risk of partner bereavement and, with higher rates of prior mental disorders, may have worse bereavement outcomes than heterosexual bereaved partners. The contribution of social factors to disenfranchised (i.e. unacknowledged) grief has been recognised. If the deceased had a stigmatised death, or the relationship to the deceased was not acknowledged, the bereaved partner's access to support is limited (Doka, 2002). However, the prevalence of poor bereavement outcomes among LGB people has not been estimated in population-based research.

Experiences of discrimination within healthcare are commonly reported among LGB people (Sinding, Barnoff, & Grassau, 2004) including at the end of life (Bristowe et al., 2018; Harding, Epiphaniou, & Chidgey-Clark, 2012), resulting in a reluctance to access healthcare and to share sexual orientation with healthcare professionals (Ramchand & Fox, 2007). A systematic review of the bereavement experiences of LGBT who had lost a partner (Bristowe, Marshall, & Harding, 2016) identified additional barriers and stressors including homophobia, failure of professionals and social networks to acknowledge the relationship, additional legal and financial issues, and the shadow of prior AIDS-related deaths.

This population-based cross-sectional survey aimed to compare the outcomes of people bereaved of a same-gender and different-gender partner.

## Methods

### Sampling

Sampling and invitations to participate were undertaken by the UK Office for National Statistics (ONS) using death registration data for England and Wales. Same-gender and different-gender participants were identified from ONS mortality data on relationship label (wife, widow, husband, widower, civil partner), gender of a decedent (male, female) and date of death. A randomly selected sample of potential participants was invited in five waves, coordinated such that the death of each invitee's partner had occurred 6 to 10 months prior to an invitation (avoiding both the most acute immediate bereavement period and the anniversary of death). Invited same-gender partners comprised all civil partners or same-gender spouses who had registered their partner's death in England and Wales between 9 September 2017 and 8 January 2018 inclusive. Different-gender (male-female) partners were randomly selected for an invitation by ONS.

The research team sent a survey pack by post to 1380 individuals who had registered the death of a different-gender civil partner or spouse and 564 individuals who had registered the death of a same-gender spouse. Survey packs included: a personalised cover letter detailing what participation would involve and how the individual could opt out of future communications; a survey booklet and freepost envelope for returning the survey; a small opt-out form (with freepost envelope); and leaflets describing bereavement support. Unique ID numbers were printed on the survey booklets, opt-out forms and cover letters, so that survey responses could be linked to further data provided by the ONS (see below) and opt-out responses could be linked to invitees. We provided URLs for electronic versions of the survey and opt-out forms so that the online response option was available. Invitees who had not participated (with the exception of those that had opted out) were sent a single reminder letter two to three weeks after the invitation.

### Sample size calculation

To estimate the prevalence of complicated grief within each group with a precision of ±5% and 95% confidence interval (CI), would require a sample of 225 same-gender participants and 225 different-gender participants, assuming a conservative estimate of 23% prevalence (Utz, Caserta, & Lund, 2011). Additionally, for a two-tailed test to detect a conservative small effect size of 0.25 in the Inventory of Complicated Grief (ICG) score between groups (80% power, 5% significance), we estimated that we would require *n* = 225 same-gender participants and *n* = 287 different-gender participants. Based on common rules of thumb for multiple linear regression (Tabachnick & Fidell, 2013), a total sample of *N* = 512 would exceed that needed to detect effects with a minimum of Cohen's *f*^2^ = 0.02 in all other planned analyses.

Due to a smaller number of potential same-gender participants in the sampling frame and difficulty in predicting response rates from both groups for this novel sampling technique, the number of invited participants from each group and number of waves were determined on a wave-by-wave basis. To do this, we projected the response rates broken down by partner gender after each wave. At the next wave we then invited all potential same-gender participants in the chosen period and then adjusted the number of different-gender participants to ensure we reached our minimum targets without excessive oversampling. We determined a priori that all responses received within 56 days (i.e. 8 weeks) of final dispatch were to be included in the final analyses.

### Measures

We collected the following self-report measures.

#### Outcomes

Grief intensity using the ICG (Prigerson et al., 1995), *α* = 0.91, and psychiatric symptoms using the 12 item General Health Questionnaire (GHQ-12) (Goldberg & Williams, 1988), *α* = 0.91. Grief intensity scores (continuous) were calculated by summing scores on the 19 items from the ICG. Psychiatric symptoms (continuous) were calculated by summing scores on the 12 items from the GHQ-12 using the 0-0-1-1 method (Goldberg & Williams, 1988). Complicated grief (binary) was defined as a score of >25 on the ICG. This is a well-validated and standardised cutoff which indicates a high risk for requiring clinical care (American Psychological Association, 2020; Mason & Tofthagen, 2019). Psychiatric caseness i.e. level of psychiatric distress that if such respondents presented in general practice, they would be likely to receive further attention (binary) was defined as a score of >2 on GHQ-12, in line with the standard cutoff (Goldberg & Williams, 1988). We measured partner gender concordance using two items ‘What is your gender?’ and ‘What was your partner or spouse's gender?’ We provided the following response options: ‘Man,’ ‘Woman,’ ‘Non-Binary (please specify the term they used),’ ‘None; I am agender,’ ‘Another gender not listed (please specify the term you use)’ and ‘Prefer not to say.’ Participants were classed as part of a different-gender couple if they and their partner were a man and a woman, and classed as a same-gender couple if they and their partner were both men or both women. Participants were also asked if they or their partner considered themselves to be transgender to characterise the sample.

#### Potential confounders

We chose the following potential confounders based on prior literature. (i) gender (binary for analysis purposes: man/woman), (ii) age (continuous: ‘How old are you?’), (iii) partner age (continuous: ‘How old was your partner or spouse when they died?’), (iv) length of relationship (continuous: ‘How many years had you been in a relationship?’), (v) ethnicity [binary for analysis purposes: minority ethnicity using categories from the UK 2011 Census ethnicity question (Office for National Statistics, 2012)], (vi) religion (binary: has religion/does not; ‘what is your religion?’: multiple response options), (vii) expectedness of death (two indicator variables: a) Less than hour's warning (including no warning) *v*. 1 h-6 months, and (b) More than 6 months' warning *v*. 1 h-6 months, based on empirical cutoffs (Carr, House, Wortman, Nesse, & Kessler, 2001); (viii) ‘How long before your partner or spouse's death did you realise that they were going to die?’ (multiple response options), (ix) traumatic life experiences [count variable: Life Events Checklist (Weathers et al., 2013), one count for each response endorsed], (x) adult bereavement (count variable: number of relationship types bereaved in adulthood); (xi) childhood bereavement (binary variable: no childhood bereavements/at least one childhood bereavement). The latter two variables were constructed from a single measure that asked the types of bereavement that participants had experienced prior to the death of their partner, providing seven options under two headings: ‘As a child (under 18)’ and ‘since turning 18’.

Potential mediator variables: We measured caregiver burden using the six item Zarit Burden Interview (ZBI-6) (Higginson, Gao, Jackson, Murray, & Harding, 2010), *α* = 0.87, social support using the Medical Outcomes Study Social Support Survey (MOS-SS) (Moser, Stuck, Silliman, Ganz, & Clough-Gorr, 2012), *α* = 0.92, loneliness using the 3-item UCLA Loneliness Scale (Hughes, Waite, Hawkley, & Cacioppo, 2004), *α* = 0.83, and discrimination using the Everyday Discrimination Scale (EDS) (Williams, Yu, Jackson, & Anderson, 1997), *α* = 0.91. Each produces a continuous score. The EDS and ZBI were recoded as binary due to non-normal distributions that could not be transformed.

#### Other variables

We measured sexual orientation using a single question with five response options, a write in (‘Other’) option and a ‘Prefer not to say’ option. The specific response options were 1 = Completely heterosexual, 2 = Mainly heterosexual, 3 = Bisexual, 4 = Mainly gay or lesbian and 5 = ‘Completely gay or lesbian’.

In line with ONS confidentiality policy, we were unable to link data to individual survey responses on the following variables, but we have included these to characterise the sample: wave of mailout, month and date of death, Index of Multiple Deprivation (IMD), cause of death, and region of registration.

### Analysis

We conducted all analyses using Stata 16. We estimated prevalence and 95% CIs for complicated grief and psychiatric caseness, comparing these in same-gender and different-gender partners. We then used multiple logistic regression to test the association of partner gender with complicated grief and psychiatric caseness (models 1a and 1b respectively), adjusting for a set of potential confounders agreed *a priori* (gender, age, partner age, length of relationship, ethnicity, religion, religiosity of partner, expectedness of death, traumatic life experiences, childhood bereavement, adult bereavement) (models 2a and 2b respectively). To avoid information loss from using binary outcome variables, and to complement our findings from logistic regressions, we also ran our models as linear regressions using continuous measures of intensity of grief and psychological distress as dependent variables. These are reported as models 1c and 1d for linear univariate regression, and 2c and 2d for multivariate models adjusted for confounders. In exploratory analyses (models 3a-3d), we then added to our regression models four potential mediator variables (caregiver burden, social support, loneliness, and discrimination) as a block adjustment to test whether these attenuated any associations between same-gender *v.* different-gender partner and the likelihood of complicated grief or psychiatric caseness.

To inform future mediation analyses in appropriate longitudinal datasets, we ran mediation models on the continuous outcomes of grief intensity and psychological distress. We tested whether there was evidence that caregiver burden, social support, loneliness, and discrimination mediate any association between same-gender *v.* different-gender bereavement. Bias-corrected bootstrapping was used to estimate CIs (Fritz & MacKinnon, 2007).

### Sensitivity analyses

As there are no standard cutoffs for ICG and GHQ-12 in our specific population, we planned sensitivity analyses with commonly used alternatives of >3 (Fridh, Rosvall, & Lindström, 2020) and >30 (Simon et al., 2011) respectively, to test whether the selection of cutoffs affected our logistic regression results. We also noted nine participants who reported they were in a same-gender relationship, but that they were either completely heterosexual or mainly heterosexual. Examination of our open field responses indicated that at least some of these participants had entered a marriage or a civil partnership for non-romantic reasons, and these participants may bias our results towards no difference between groups. To test whether these participants affected results, sensitivity analyses were performed with them excluded. We report methods and findings in line with the STROBE statement on cross-sectional design.

#### Ethics statement

Approval was provided by King's College London Research Ethics Committee (RESCM-17/18-5668). Participants were sent a cover letter with information about the study's purpose, the voluntary nature of participation, how to participate, opt out or withdraw, and whom to contact if they had any queries. Participants consented to the study by returning the completed paper or online survey. To preserve anonymity of those wishing to participate without disclosing their identity, no additional written consent was required. However, individuals were asked to provide contact details if they were willing to be contacted in the future, participate in a subsequent qualitative interview, or to receive a summary of findings. The survey included two measures [ICG (Prigerson et al., 1995) and GHQ-12 (Goldberg & Williams, 1988)] that, above a specific screening threshold for each, would suggest complicated grief and likely psychiatric disorder respectively. Where a participant's responses reached either threshold (and they had provided contact details) the researchers contacted them to suggest possible sources of support.

#### Role of the funding source

The funder had no role in the collection, analysis, or interpretation of data; in the writing of the report; or in the decision to submit the paper for publication.

## Results

### Sample characteristics

In total, 569/1945 responses to the survey were received, with an overall response rate of 29.3%. There was an approximate response rate of 41.3% for same-gender partners and 23.8% for different-gender partners. This rate is approximate as some participants were transgender: gender on death registration, which was used to invite potential participants, does not always correspond with self-reported gender identity, which was used to classify participants in our sample. Participants resided in regions across England and Wales, with only 76 residing in London and 115 in South East England. The cause of partner death was most commonly cancer (*n* = 278, 48.9%). The sample had a mean IMD of 3.4 (s.d. = 1.35).

One hundred and fifty men participating were bereaved of a male partner, 76 men of a female partner, 78 women of a female partner, and 253 women of a male partner. This made for 233 same-gender partners and 329 mixed gender partners. Three participants were transgender. The sample had a mean age of 67.5 (s.d. = 11.4). The single non-binary participant was excluded from analyses. Regarding sexual orientation, 318 participants identified as completely or mainly heterosexual, 16 as bisexual, and 204 participants as completely or mainly lesbian/gay. In terms of ethnicity, 537 participants identified as white and 15 as from a minority ethnic group. Further participant characteristics are described in Table 1.

**Table 1.** Sample characteristics

	Full sample	Same-gender partner	Different-gender partner
*n*	568	233	329
Age – mean (s.d.)	67.5 (11.4)	63.6 (11.3)	70.2 (10.7)
Gender			
Woman	334	78	253
Man	231	155	76
Sexual orientation			
Completely Hetero	319	5	312
Mainly hetero	9	4	5
Bisexual	16	15	1
Mainly gay/lesbian	31	31	0
Completely gay/lesbian	173	173	0
Gender modality			
Cisgender	488	209	277
Transgender	3	2	1
Race			
White	537	218	317
BAME	15	7	8
Region			
East of England	53		
East Midlands	38		
London	76		
North East England	20		
North West England	58		
South East England	115		
Wales	32		
West Midlands	49		
Yorkshire and the Humber	39		
Other/unclear	19		
Cause of Death			
Pneumonia	15		
Cerebro-vascular disease	25		
Ischaemic heart disease	51		
Other circulatory diseases	39		
Respiratory diseases	28		
Dementia	37		
Cancer	278		
Other diseases	96		

BAME, Black, Asian, and minority ethnic.

*Note.* Discrepancies in totals are due to missing data. Data not split by gender could not be linked to our gender data in line with ONS policy.

### Missing data

Missing data on the 19 items from the ICG ranged from 0.00% to 1.41% and on the 12 GHQ-12 items from 1.05% to 2.28%. Due to these low levels of missing data and no discernible patterns of missingness, we replaced missing values for these items using stochastic regression imputation with the respective measures' non-missing items as the only predictors.

Missing data on other variables ranged from 0.35% (for child bereavement and adult bereavement) to 6.34% (for social support). Little's test was performed and indicated that the missingness was not associated with the observed data (Little, 1988). Thus, missing data of measures other than the ICG and GHQ-12 were handled using multiple imputations with 20 imputations. Results were pooled by Rubin's combination rules for all analyses (Rubin, 2004), with the exception of mediation modelling, which was pooled using means due to statistical software limitations.

### Prevalence of complicated grief and psychiatric caseness

We determined that 66.1%, [95% CI (60.0–72.2)], of participants bereaved of a same-gender partner and 59.2%, [95% CI (53.9–64.6)], of participants bereaved of a different-gender partner reached the ICG threshold for complicated grief. We determined that 76.0% [95% CI (70.5–81.5)] of participants bereaved of a same-gender partner and 69.3% [95% CI (64.3–74.3)] of participants bereaved of a different-gender partner exceeded the threshold for psychiatric caseness based on GHQ-12 scores.

### Relationships of same-gender *v.* different-gender partner bereavement with complicated grief and psychiatric caseness

When adjusting logistic regression models for possible confounders, being bereaved of a same-gender partner was not significantly associated with a higher odds of having complicated grief [OR  1.56, 95% CI (0.98–2.47), *p* = 0.059 (see Table 2). However, being bereaved of a same-gender partner was significantly associated with higher odds of psychiatric caseness [OR 1.66, 95% CI (1.02, 2.71), *p* = 0.043].

**Table 2.** Odds ratios, 95% CIs for logistic regressions

	Complicated Grief caseness		Psychiatric caseness	
	OR [95% CI]	*p*	*OR* [95% CI]	*p*
Bivariate association (unadjusted associations)				
Model 1a			Model 1b	
Same-gender partner	1.34 [0.95–1.90]	0.098	1.42 [0.97–2.08]	0.069
	aOR [95% CI]	*p*	aOR [95% CI]	*p*
Adjusted for potential confounders^a^ (final model)				
Model 2a		Model 2b		
Same-gender partner	1.56 [0.98–2.47]	0.059	***1***.***67 [1***.***02–2***.***71]***	***0***.***043***
Adjusted for potential confounders and potential mediators^b^				
Model 3a		Model 3b		
Same-gender partner	1.28 [0.76–2.15]	0.363	1.36 [0.79–2.34]	0.269

aOR, adjusted Odds Ratio; ZBI, Zarit Burden Inventory.

*Note.* Bolded Effects are statistically significant, sensitivity analysis indicated that italicised effects were non-significant when those whose partner's gender is unintuitive for their reported sexual orientation are excluded.

a Final models: adjusted for gender, age, partner age, length of relationship, ethnicity, religion, religiosity of partner, expectedness of death, traumatic life experiences, childhood bereavement, adult bereavement.

b Adjusting final models for potential mediators: care-giver burden (Zarit Burden Interview; binary), discrimination (binary), social support (continuous), loneliness (continuous)

**Table 3.** Betas, 95% CIs for linear regressions

	Grief Intensity		Psychiatric Symptoms	
	*B* [95% CI]	*p*	*B* [95% CI]	*p*
Bivariate association (unadjusted associations)				
Model 1c		Model 1d		
Same-gender Partner	1.16 [−1.14 to 3.46]	0.321	**1**.**07 [0**.**39–1**.**77]**	**0**.**002**
Model 2c		Model 2d		
Adjusted for potential confounders^a^ (final model)				
Model 2c		Model 2d		
Same-gender Partner	1.86 [−0.91 to 4.63]	0.188	**1**.**54 [0**.**69–2**.**40]**	**<0.001**
Adjusted for potential confounders and potential mediators^b^				
Model 3c		Model 3d		
Same-gender Partner	−0.07 [−2.43 to 2.28]	0.951	**0**.**99 [0**.**26–1**.**73]**	**0**.**008**

ZBI, Zarit Burden Inventory.

Bolded Effects are statistically significant, sensitivity analyses indicated that effects were not changed by the inclusion of participants whose partner's gender is unintuitive for their reported sexual orientation are excluded.

a Final models: adjusted for gender, age, partner age, length of relationship, ethnicity, religion, religiosity of partner, expectedness of death, traumatic life experiences, childhood bereavement, adult bereavement.

b adjusting final models for potential mediators: care-giver burden (Zarit Burden Interview; binary), discrimination (binary), social support (continuous), loneliness (continuous).

When adding potential mediators, partner gender was not significantly associated with complicated grief, OR 1.28, 95% CI [0.76–2.15], *p* = 0.363 or psychiatric caseness, OR 1.36, 95% CI [0.78–2.33], *p* = 0.269.

### Relationships of same-gender *v.* different-gender partner bereavement with grief intensity and levels of psychiatric symptoms

When adjusting linear regression models for possible confounders (Table 3), same-gender partners did not differ significantly in grief intensity from different-gender partners [*B* = 1.86, 95% CI (−0.091 to 4.63), *p* = 0.192] but were significantly more likely to screen positive for psychiatric symptoms [*B* = 1.54, 95% CI (0.69–2.40), *p* < 0.001].

When adding potential mediators, the association between partner gender and grief intensity was attenuated and remained non-significant [*B* = −0.07, 95% CI (−2.43 to 2.28), *p* = 0.951], while the association between partner gender and number of psychiatric symptoms attenuated but remained significant [*B* = 0.99, 95% CI (0.26–1.73), *p* = 0.008].

### Mediation model using cross-sectional data

We found evidence to support an indirect mediating effect of loneliness in the association between same *v.* different-gender partner bereavement and grief intensity (continuous), and an indirect mediating effect of social support, loneliness and caregiver burden in the association between same *v.* different-gender partner bereavement and psychiatric symptoms (Table 4). No other significant mediating effects were found.

**Table 4.** Exploratory Analyses to test for potential mediation

Mediator	Grief intensity	Psychiatric symptoms
	Indirect effect [95% CI]	Indirect effect [95% CI]
Social Support	−0.05 [−0.20 to 0.06]	−**0**.**37 [**−**1**.**03 to −0**.**01]**
Loneliness	**0**.**45 [0**.**05–0**.**79]**	**1**.**60 [0**.**16–2**.**81]**
Everyday Discrimination	−0.02 [−0.15 to 0.08]	−0.03 [−0.34 to 0.13]
Caregiver Burden (Zarit Burden Inventory)	0.01 [−0.05 to 0.09]	**0**.**44 [0**.**11–0**.**98]**
Total Effect	0.39 [−0.09 to 0.76]	**1.64 [0**.**13–2**.**75]**

Bolded numbers are for statistically significant findings.

### Sensitivity analyses

Sensitivity analyses indicated that excluding participants bereaved of partners whose gender did not coincide with the respondent's sexual orientation resulted in partner gender no longer being associated with psychiatric caseness in logistic regression model 2.

Sensitivity analyses changing the threshold for the ICG did not change the pattern of associations with partner gender. When changing the cutoff for the GHQ-12 [from >2 to >3, (Fridh et al., 2020)] in the GHQ-12 being positively associated with psychiatric caseness in our bivariate (unadjusted) analysis. Sensitivity analyses otherwise did not change the pattern of associations with partner gender.

## Discussion

This is the first study to compare bereavement outcomes for partners of same-gender and different-gender decedents using representative population-based sampling. Although these groups did not significantly differ in their likelihood of reaching the threshold for complicated grief or in grief intensity, they did differ in terms of psychiatric caseness and number of psychological symptoms, such that worse outcomes were seen in people bereaved of a same-gender partner than people bereaved of a different-gender partner. This suggests that psychological morbidity is worse in people bereaved of a same-gender partner, but that the proportions who meet the criteria for complicated grief are either equal or any differences are too small to detect in a sample of this size. Notably, examination of our CIs and point estimates suggests our data favour higher proportions of complicated grief in same-gender partners over no difference (Greenland, 2012), but further research in larger longitudinal samples will be required to adequately explore these findings.

It is notable that high proportions of study participants met thresholds for psychiatric caseness 6–10 months after the death of their partner. For the GHQ, 76.0% of same-gender and 63.3% of different-gender participants met the caseness threshold. For the ICG, this was 66.1% and 59.2% respectively. There is a lack of population-based data for comparison of both outcomes, with prior studies having sampled highly specific populations (e.g. by cause of death, those self-identifying with bereavement needs, prior mental health concerns, ethnicity), or pooled bereavement types (spouse, sibling, parent, friend etc).

In our mediation analyses we found an indirect mediating effect of loneliness in the association between same *v.* different-gender partner bereavement and grief intensity (continuous), and a similar effect of social support, loneliness and caregiver burden for psychiatric symptoms. This tentative evidence supports the model of bereavement for LGBT+ people (Bristowe et al., 2016). The model was developed from a synthesis of the available evidence, which identified deficiencies in familial and community support as a challenge more frequently experienced by this population. In addition, poor experiences of care at the end-of-life worsened bereavement outcomes.

It should also be noted that LGB people generally have worse psychological symptoms than heterosexual people (Pitman et al., 2021) and this is generally attributed to a stigmatising social environment (Meyer, 2003), even in contexts like England and Wales where same-sex marriage is legal and discrimination on the basis of sexual orientation is not. Thus, our observed differences could have been due to pre-bereavement sociocultural factors, rather than differences in the bereavement process *per se*. However, our exploratory mediation analyses suggest that bereavement-related factors may play a role. Regardless, our results suggest that the mental health disparities described above are also apparent when faced with an additional major stressor such as bereavement, which would indicate a need for additional support for bereaved LGB people either way. Furthermore, as a legal relationship is typically required to report the death of a partner in England and Wales, we expect that our LGB sample would primarily consist of those who were open enough about their relationship to access marriage or a civil partnership and felt empowered to participate. The fact that we found group differences even in this group is striking.

There are several limitations to this study. We acknowledge the low participation of non-white bereaved people. In relation to this we note the findings of two nationally representative surveys of common mental disorders among LGB adults in England (Pitman et al., 2021). The study reported that those who identified their sexual orientation as ‘other’ were more likely to be non-white, had a high prevalence of the common mental disorder, and to have a religious affiliation. It is possible that these individuals were less likely to register the death as same-gendered partner of a decedent or to participate in our survey. There may also have been non-response bias in our study among those with less complex grief. We had a small number of respondents who identified as bisexual (*n* = 16), and were unable to determine whether bisexuality influenced outcomes, as prior data has found bisexual individuals are at greater risk of poor mental health than lesbians and gay men (Chan, Operario, & Mak, 2020). Due to the block adjustment, the analysis cannot identify the variables associated with outcomes. We also acknowledge that any attenuation when adding potential mediators could be indicative of mediation, but were by definition unable to conduct formal mediation analysis using cross-sectional data (Kline, 2015). Our study was underpowered to stratify by gender, but further quantitative and qualitative work is needed to understand how bereavement experiences might differ in female-female partnerships as compared to male-male partnerships. Further, although the prior review suggested similar bereavement challenges and outcomes for trans people (Bristowe et al., 2016), we were unable to investigate their outcomes in this study due to the limitations of death registration data.

Given the higher prevalence of psychological distress among same-gender bereaved partners, care prior to death must be inclusive and supportive of same-gender partners (Bristowe et al., 2018). Perceived poor quality of patient care is predictive of worse caregiver outcomes post-death (Garrido & Prigerson, 2014). Given the evidence for lower access to, and satisfaction with, healthcare services reported by LGB people (Elliott et al., 2015), attention should be paid to screening and support pre- and post-bereavement to identify those most at risk of poor outcomes in order to provide early and appropriate intervention. Simple guidance exists to ensure inclusive practice for LGBT patients and significant others at the end of life and facing an expected death, and should be implemented (Bristowe et al., 2018). Similar guidance that focuses on language, communication, environment (e.g. use of visible markers of inclusion) and routine collection of data on gender and sexual orientation should be developed for bereavement services to be inclusive of those who experience the unexpected death of a partner.

## Data

De-identified participant data will be made available from the corresponding author after approval of a proposal with a signed data access agreement.

## Supplementary material

For supplementary material accompanying this paper visit https://doi.org/10.1017/S0033291722000496. click here to view supplementary material

## References

[ref1] American Psychological Association. (2020). Inventory of Complicated Grief (ICG). Retrieved from https://www.apa.org/pi/about/publications/caregivers/practice-settings/assessment/tools/complicated-grief.

[ref2] Bristowe, K., Hodson, M., Wee, B., Almack, K., Johnson, K., Daveson, B. A., … Harding, R. (2018). Recommendations to reduce inequalities for LGBT people facing advanced illness: ACCESSCare national qualitative interview study. Palliative Medicine, 32(1), 23–35. doi: 10.1177/0269216317705102. 28502218 PMC5758934

[ref3] Bristowe, K., Marshall, S., & Harding, R. (2016). The bereavement experiences of lesbian, gay, bisexual and/or trans* people who have lost a partner: A systematic review, thematic synthesis and modelling of the literature. Palliative Medicine, 30(8), 730–744. doi: 10.1177/0269216316634601. 26944532 PMC4984311

[ref4] Carr, D., House, J. S., Wortman, C., Nesse, R., & Kessler, R. C. (2001). Psychological adjustment to sudden and anticipated spousal loss among older widowed persons. The Journals of Gerontology Series B: Psychological Sciences and Social Sciences, 56(4), S237–S248. 11445616 10.1093/geronb/56.4.s237

[ref5] Chan, R. C. H., Operario, D., & Mak, W. W. S. (2020). Bisexual individuals are at greater risk of poor mental health than lesbians and gay men: The mediating role of sexual identity stress at multiple levels. Journal of Affective Disorders, 260, 292–301. doi: 10.1016/j.jad.2019.09.020. 31521866

[ref6] Doka, K. E. (2002). Disenfranchised grief: New directions, challenges, and strategies for practice. Champaign, IL: Research Press.

[ref7] Elliott, M. N., Kanouse, D. E., Burkhart, Q., Abel, G. A., Lyratzopoulos, G., Beckett, M. K., … Roland, M. (2015). Sexual minorities in England have poorer health and worse health care experiences: A national survey. Journal of General Internal Medicine, 30(1), 9–16. doi: 10.1007/s11606-014-2905-y. 25190140 PMC4284269

[ref8] Elwert, F., & Christakis, N. (2008). The effect of widowhood on mortality by the causes of death of both spouses. American Journal of Public Health, 98(11), 2092–2098. 18511733 10.2105/AJPH.2007.114348 PMC2636447

[ref9] Flentje, A., Heck, N. C., Brennan, J. M., & Meyer, I. H. (2020). The relationship between minority stress and biological outcomes: A systematic review. Journal of Behavioral Medicine, 43(5), 673–694. doi: 10.1007/s10865-019-00120-6. 31863268 PMC7430236

[ref10] Fridh, M., Rosvall, M., & Lindström, M. (2020). Poor psychological health and 5-year suicide mortality: A population-based prospective cohort study. Social Science and Medicine, 258, 113056. 32516638 10.1016/j.socscimed.2020.113056

[ref11] Fritz, M. S., & MacKinnon, D. P. (2007). Required sample size to detect the mediated effect. Psychological Science, 18(3), 233–239. doi: 10.1111/j.1467-9280.2007.01882.x. 17444920 PMC2843527

[ref12] Garrido, M. M., & Prigerson, H. G. (2014). The end-of-life experience: Modifiable predictors of caregivers’ bereavement adjustment. Cancer, 120(6), 918–925. doi: 10.1002/cncr.28495. 24301644 PMC3947659

[ref13] Goldberg, D., & Williams, P. (1988). A user's guide to the general health questionnaire. Windsor: NFER-Nelson.

[ref14] Greenland, S. (2012). Nonsignificance plus high power does not imply support for the null over the alternative. Annals of Epidemiology, 22(5), 364–368. 10.1016/j.annepidem.2012.02.007. 22391267

[ref15] Harding, R., Epiphaniou, E., & Chidgey-Clark, J. (2012). Needs, experiences, and preferences of sexual minorities for end-of-life care and palliative care: A systematic review. Journal of Palliative Medicine, 15(5), 602–611. doi: 10.1089/jpm.2011.0279. 22401314

[ref16] Higginson, I. J., Gao, W., Jackson, D., Murray, J., & Harding, R. (2010). Short-form Zarit Caregiver Burden interviews were valid in advanced conditions. Journal of Clinical Epidemiology, 63(5), 535–542. doi: 10.1016/j.jclinepi.2009.06.014. 19836205

[ref17] Hughes, M. E., Waite, L. J., Hawkley, L. C., & Cacioppo, J. T. (2004). A short scale for measuring loneliness in large surveys: Results from two population-based studies. Research on Aging, 26(6), 655–672. 18504506 10.1177/0164027504268574 PMC2394670

[ref18] Jackson, S. E., Brown, J., Grabovac, I., Cheeseman, H., Osborne, C., & Shahab, L. (2020). Smoking and quitting behavior by sexual orientation: A cross-sectional survey of adults in England. Nicotine & Tobacco Research, 23(1), 124–134. doi: 10.1093/ntr/ntaa042. PMC7789956 32115647

[ref19] Kline, R. B. (2015). The mediation myth. Basic and Applied Social Psychology, 37(4), 202–213. doi: 10.1080/01973533.2015.1049349.

[ref20] Little, R. J. (1988). A test of missing completely at random for multivariate data with missing values. Journal of the American Statistical Association, 83(404), 1198–1202.

[ref21] Machalek, D. A., Poynten, M., Jin, F., Fairley, C. K., Farnsworth, A., Garland, S. M., … Grulich, A. E. (2012). Anal human papillomavirus infection and associated neoplastic lesions in men who have sex with men: A systematic review and meta-analysis. The Lancet Oncology, 13(5), 487–500. 10.1016/S1470-2045(12)70080-3. 22445259

[ref22] Mason, T. M., & Tofthagen, C. S. (2019). Complicated grief of immediate family caregivers: A concept analysis. Advances in Nursing Science, 42(3), 255–265. 30531354 10.1097/ANS.0000000000000243

[ref23] Meads, C., Martin, A., Grierson, J., & Varney, J. (2018). Systematic review and meta-analysis of diabetes mellitus, cardiovascular and respiratory condition epidemiology in sexual minority women. BMJ Open, 8(4), e020776. doi: 10.1136/bmjopen-2017-020776. PMC5905763 29666136

[ref24] Meads, C., & Moore, D. (2013). Breast cancer in lesbians and bisexual women: Systematic review of incidence, prevalence and risk studies. BMC Public Health, 13(1), 1127. doi: 10.1186/1471-2458-13-1127. 24313963 PMC3890640

[ref25] Mercer, C. H., Prah, P., Field, N., Tanton, C., Macdowall, W., Clifton, S., … Sonnenberg, P. (2016). The health and well-being of men who have sex with men (MSM) in Britain: Evidence from the third National Survey of Sexual Attitudes and Lifestyles (Natsal-3). BMC Public Health, 16, 525–525. doi: 10.1186/s12889-016-3149-z. 27386950 PMC4936006

[ref26] Meyer, I. H. (2003). Prejudice, social stress, and mental health in lesbian, gay, and bisexual populations: Conceptual issues and research evidence. Psychological Bulletin, 129(5), 674–697. 10.1037/0033-2909.129.5.674. 12956539 PMC2072932

[ref27] Moser, A., Stuck, A., Silliman, R., Ganz, P., & Clough-Gorr, K. (2012). The eight-item modified medical outcomes study social support survey: Psychometric evaluation showed excellent performance. Journal of Clinical Epidemiology, 65(10), 1107–1116. 22818947 10.1016/j.jclinepi.2012.04.007 PMC4119888

[ref28] Office for National Statistics. (2012). Ethnicity and National Identity in England and Wales 2011. Retrieved from Office for National Statistics website: https://www.ons.gov.uk/peoplepopulationandcommunity/culturalidentity/ethnicity/articles/ethnicityandnationalidentityinenglandandwales/2012-12-11.

[ref29] Pitman, A., Marston, L., Lewis, G., Semlyen, J., McManus, S., & King, M. (2021). The mental health of lesbian, gay, and bisexual adults compared with heterosexual adults: Results of two nationally representative English household probability samples. Psychological Medicine, 1–10. doi: 10.1017/S0033291721000052. 33592165

[ref30] Prigerson, H., Maciejewski, P., Reynolds, C., Bierhals, A., Newsom, J., Fasiczka, A., … Miller, M. (1995). The inventory of complicated grief: A scale to measure maladaptive symptoms of loss. Psychiatry Research, 59(1–2), 65–79. 8771222 10.1016/0165-1781(95)02757-2

[ref31] Prigerson, H., Silverman, G., Jacobs, S., Maciejewski, P., Kasl, S., & Rosenheck, R. (2001). Traumatic grief, disability and the underutilization of health services: A preliminary look. Primary Psychiatry, 8(1–2), 66–69.

[ref32] Quinn, G. P., Sanchez, J. A., Sutton, S. K., Vadaparampil, S. T., Nguyen, G. T., Green, B. L., … Schabath, M. B. (2015). Cancer and lesbian, gay, bisexual, transgender/transsexual, and queer/questioning (LGBTQ) populations. CA: A Cancer Journal for Clinicians, 65(5), 384–400. doi: 10.3322/caac.21288. 26186412 PMC4609168

[ref33] Ramchand, R., & Fox, C. (2007). Access to optimal care among gay and bisexual men: Identifying barriers and promoting culturally competent care. In R. Wolitski, R. Stall, & R. Valdiserri (Eds.), Unequal opportunity: Health disparities affecting gay and bisexual men in the United States (pp. 355–378). New York: Oxford University Press.

[ref34] Robinson, K., Galloway, K., Bewley, S., & Meads, C. (2017). Lesbian and bisexual women's gynaecological conditions: A systematic review and exploratory meta-analysis. BJOG: An International Journal of Obstetrics & Gynaecology, 124(3), 381–392. doi: 10.1111/1471-0528.14414. 27862853 PMC5363366

[ref35] Rubin, D. B. (2004). Multiple imputation for nonresponse in surveys (Vol. 81). New Jersey: John Wiley & Sons.

[ref36] Saunders, C. L., Meads, C., Abel, G. A., & Lyratzopoulos, G. (2017). Associations between sexual orientation and overall and site-specific diagnosis of cancer: Evidence from two national patient surveys in England. Journal of Clinical Oncology: Official Journal of the American Society of Clinical Oncology, 35(32), 3654–3661. doi: 10.1200/JCO.2017.72.5465. 28945501 PMC5855217

[ref37] Shokoohi, M., Salway, T., Ahn, B., & Ross, L. E. (2020). Disparities in the prevalence of cigarette smoking among bisexual people: A systematic review, meta-analysis and meta-regression. Tobacco Control, 30, e78–e86. doi: 10.1136/tobaccocontrol-2020-055747. 32934092

[ref38] Simon, N. M., Wall, M. M., Keshaviah, A., Dryman, M. T., LeBlanc, N. J., & Shear, M. K. (2011). Informing the symptom profile of complicated grief. Depression and Anxiety, 28(2), 118–126. 21284064 10.1002/da.20775 PMC3079952

[ref39] Sinding, C., Barnoff, L., & Grassau, P. (2004). Homophobia and heterosexism in cancer care: The experiences of lesbians. Canadian Journal of Nursing Research, 36(4), 170–188. 15739943

[ref40] Stephen, A., MacDuff, C., Petrie, D., Tseng, F.-M., Schut, H., Skar, S., … Wilson, S. (2015). The ecomonic cost of bereavement in Scotland. Death Studies, 39(3), 151–157. 25255790 10.1080/07481187.2014.920435

[ref41] Stroebe, M., Schut, H., & Stroebe, W. (2007). Health outcomes of bereavement. Lancet (London, England), 370(9603), 1960–1973. doi: 10.1016/S0140-6736(07)61816-9. 18068517

[ref42] Tabachnick, B. G., & Fidell, L. S. (2013). Using multivariate statistics (6th ed.). New York: Pearson.

[ref43] Thompson, L., Breckenridge, J., Gallagher, D., & Peterson, J. (1984). Effects of bereavement on self-perceptions of physical health in elderly widows and widowers. Journal of Gerontology, 39, 309–14. 6715808 10.1093/geronj/39.3.309

[ref44] Utz, R., Caserta, M., & Lund, D. (2011). Grief, depressive symptoms, and physical health among recently bereaved spouses. The Gerontologist, 52(4), 460–471. 22156713 10.1093/geront/gnr110 PMC3391379

[ref45] Weathers, F., Blake, D., Schnurr, P., Kaloupek, D., Marx, B., & Keane, T. (2013). The life events checklist for DSM-5 (LEC-5*)*. White River Junction, VT: National Center for PTSD.

[ref46] Williams, D., Yu, Y., Jackson, J., & Anderson, N. (1997). Racial differences in physical and mental health: Socioeconomic status, stress and discrimination. Journal of Health Psychology, 2(3), 335–351. 22013026 10.1177/135910539700200305

