# Supplementary material for: Does the impact of bereavement vary between same and different gender partnerships? A representative national, cross-sectional study
Source: Psychol Med. 2022 May 27;53(9):3849–57. doi: 10.1017/S0033291722000496 (PMC10317796; doi:10.1017/S0033291722000496)
Supplement: Supplementary file 1 [file S0033291722000496sup001.docx]

| Online Table S1 Odds Ratios, 95% Confidence Intervals for Logistic Regressions with Same-Gender Partners Only | | | | | |
| --- | --- | --- | --- | --- | --- |
|  | Complicated Grief caseness | | | Psychiatric caseness | |
|  | *aOR* [95% CI] | *p* | | *aOR* [95% CI] | *p* |
| Bivariate association (unadjusted association) | | | | | |
| Model 1a | | | Model 1b | | |
| Female | 1·48 [0·82, 2·67] | 0·193 | | **2·87 [1·36, 6·06]** | **0·006** |
|  | *aOR* [95% CI] | *p* | | *aOR* [95% CI] | *p* |
| Adjusted for potential confounders* (final model) | | | | | |
| Model 2a | | | Model 2b | | |
| Female | 1·56 [0·81, 3·01] | 0·185 | | **3**·**13 [1**·**41, 6**·**96]** | **0**·**005** |
| Adjusted for potential confounders and potential mediators** | | | | | |
| Model 3a | | | Model 3b | | |
| Female | 1·64 [0·72, 3·70] | 0·236 | | **3·50 [1·34, 9·13]** | **0·010** |
| *Note:* aOR = adjusted Odds Ratio. ZBI = Zarit Burden Inventory. Bolded Effects are statistically significant.  * Final models: adjusted for gender, age, partner age, length of relationship, ethnicity, religion, religiosity of partner, expectedness of death, traumatic life experiences, childhood bereavement, adult bereavement.  ** adjusting final models for potential mediators: care-giver burden (Zarit Burden Interview; binary), discrimination (binary), social support (continuous), loneliness (continuous) | | | | | |

| Online Table S2 Betas, 95% Confidence Intervals for Linear Regressions with Same-Gender Partners Only | | | | | |
| --- | --- | --- | --- | --- | --- |
|  | Grief Intensity | | | Psychiatric Symptoms | |
|  | *B* [95% CI] | *p* | | *B* [95% CI] | *p* |
| Bivariate association (unadjusted association) | | | | | |
| Model 1c | | | Model 1d | | |
| Female | 2·29 [-1·39, 5·96] | 0·222 | | **2**·**14 [1**·**02, 3**·**26]** | **<0·001** |
| Model 2c | | | Model 2d | | |
| Adjusted for potential confounders* (final model) | | | | | |
| Model 2c | | | Model 2d | | |
| Female | 2·54 [-1·10, 6·18] | 0·170 | | **2**·**24 [1**·**09, 3**·**38]** | **<0·001** |
| Adjusted for potential confounders and potential mediators** | | | | | |
| Model 3c | | | Model 3d | | |
| Female | 2·02 [-1·06, 5·09] | 0·197 | | **1**·**92 [0**·**96, 2**·**88]** | **<0·001** |
| ZBI = Zarit Burden Inventory. Bolded Effects are statistically significant.  * Final models: adjusted for gender, age, partner age, length of relationship, ethnicity, religion, religiosity of partner, expectedness of death, traumatic life experiences, childhood bereavement, adult bereavement.  ** adjusting final models for potential mediators: care-giver burden (Zarit Burden Interview; binary), discrimination (binary), social support (continuous), loneliness (continuous) | | | | | |
